# Oral Immunotherapy and Basophil and Mast Cell Reactivity in Food Allergy

**DOI:** 10.3389/fimmu.2020.602660

**Published:** 2020-12-14

**Authors:** Anuya Paranjape, Mindy Tsai, Kaori Mukai, Ramona A. Hoh, Shilpa A. Joshi, R. Sharon Chinthrajah, Kari C. Nadeau, Scott D. Boyd, Stephen J. Galli

**Affiliations:** ^1^Department of Pathology, Stanford University School of Medicine, Stanford, CA, United States; ^2^Sean N. Parker Center for Allergy and Asthma Research, Stanford University School of Medicine, Stanford, CA, United States; ^3^Division of Pulmonary, Allergy and Critical Care Medicine and Division of Allergy, Immunology and Rheumatology, Stanford University, Stanford University School of Medicine, Stanford, CA, United States; ^4^Department of Microbiology and Immunology, Stanford University School of Medicine, Stanford, CA, United States

**Keywords:** food oral immunotherapy, basophil activation tests, mast cells, skin prick tests, IgE, IgG, B cells

## Abstract

Basophil activation tests (BATs) can closely monitor, *in vitro*, a patient’s propensity to develop type I hypersensitivity reactions. Because of their high specificity and sensitivity, BATs have become promising diagnostic tools, especially in cases with equivocal clinical histories, skin prick test results, and/or levels of specific IgE to allergen extracts. BATs also are useful as tools for monitoring the effects of treatment, since oral immunotherapy (OIT) studies report a diminution in patients’ basophil responsiveness over the course of OIT. This review will discuss the BAT findings obtained before, during, and after OIT for food allergy. We will mainly focus on the association of basophil responsiveness, and alterations in basophil surface markers, with clinical outcomes and other clinical features, such as blood levels of specific IgG and IgE antibodies. The detailed analysis of these correlations will ultimately facilitate the use of BATs, along with other blood biomarkers, to differentiate short-term desensitization versus sustained unresponsiveness and to improve treatment protocols. Given the critical anatomic location of mast cells adjacent to the many IgE^+^ plasma cells found in the gastrointestinal tissues of allergic individuals, we will also discuss the role of gastrointestinal mast cells in manifestations of food allergies.

## Introduction

Human and mouse studies have shown that mast cells and basophils are the primary immune effector cells in IgE-mediated food allergy (1–4). Most commonly, food allergy manifests as a form of immediate hypersensitivity, in which engagement of IgE bound to FcϵRI on mast cells and basophils by specific food allergens leads to the release of pre-formed and newly synthesized mediators that elicit a range of pathological responses in several target tissues. Such responses range from hives, itching, mild gastrointestinal discomfort, and diarrhea to intense systemic reactions which, in some cases, result in rapidly fatal anaphylaxis (5, 6).

Although FcϵRI are highly expressed on both mast cells and basophils, these effectors are distinct cell populations that are regulated by different transcription factors, express distinct cell surface receptors, reside in anatomically distinct locations (7–10) and exhibit different activation thresholds to IgE-dependent stimulation, including that mediated by food allergens (11). In mouse models of food allergy, TSLP-elicited basophil expansion appears to be pivotal for cutaneous sensitization with food allergens (12–14) whereas IL-9-producing mucosal mast cells appear to be critical for intestinal mastocytosis after intragastric allergen exposure (3). In humans, studies of cat and peanut allergies have indicated that anti-IgE treatments might exhibit different response kinetics in skin mast cells and blood basophils (15, 16). It therefore should be kept in mind that basophils and mast cells may have complementary but distinct roles in the context of mouse or human food allergy.

This review will focus on basophil activation tests (BATs), which directly assess basophil reactivity, and skin prick tests (SPTs), which assess skin mast cell reactivity, obtained during food oral immunotherapy (OIT). We will discuss these in the context of clinical outcomes and will consider the use of these tools in monitoring OIT treatments.

## Basophil Activation Tests (BATs)

BATs are *ex vivo* flow cytometry-based assays for measuring basophil activation. In 1991, Knol et al. noted increased expression of CD63 on the plasma membrane of purified basophils following their activation with anti-IgE or fMLP and also found the close correlation of increased basophil expression of CD63 with histamine release (17). The development of flow cytometry-based techniques to gate on human blood basophils made the assessment of CD63 possible in whole blood without any purification step (18, 19). Subsequent studies established the reliability of another activation maker, CD203c (20, 21), but differences in the activation kinetics of CD203c vs CD63 pointed towards differences in their mechanisms of up-regulation (22). While CD63 and CD203c remain the most popular markers, many other activation markers, such as CD107a, CD13, CD164, CD69, CD11b, and diamine oxidase (23, 24), have been used for assessment of basophil activation (25–27). The pros and cons of various basophil gating strategies and activation markers have been discussed in detail elsewhere (27–29). Because of their specificity and sensitivity, BATs are being evaluated for the diagnosis of food, drug, and venom allergies, and for monitoring the effects of immunotherapy or the natural resolution of allergies (27–30).

However, BAT studies have differed in anticoagulants used for blood collection, temperature and duration of blood storage before the test, and the presence or absence of IL-3 priming (21, 31, 32). Mukai et al. compared CD63 and CD203c expression at baseline and post activation among four conditions of storage (at room temperature for 4 or 24 h and at 4°C for 4 or 24 h) using blood collected in either EDTA or heparin (33). Activation-induced CD63 upregulation cannot be noted in blood samples collected in EDTA, emphasizing the need for extracellular physiological calcium/magnesium for CD63 upregulation (34). However, blood collected in heparin yielded similar outputs in CD63 and CD203c upregulation 4 or 24 h post blood draw, if stored at 4°C (33).

Another major difference in BAT protocols is the use of whole blood or enriched preparations of basophils (35). Whole blood preparations not only better mirror the physiological or pathological *in vivo* conditions [e.g., the presence of soluble factors and blocking antibodies (26, 32, 36, 37)], but also allow insight into resting levels of activation marker expression (26). However, since CD63 is a non-exclusive marker for basophils, there have been concerns that platelets binding to basophils might falsely increase “basophil CD63 expression” in whole blood assays (38, 39). Importantly, using flow cytometry and an immunohistochemical staining analysis with the platelet-specific marker CD41, Mukai et al. found that the appearance of CD63^hi^ basophils is primarily due to basophil-derived CD63 (33).

An alternate approach to conventional BATs is the use of fluorescent avidin. Positively-charged avidin binds to negatively-charged granule constituents that are exteriorized on the cell surface post activation (39). While this method is relatively new, it holds the promise of offering a more sensitive and specific method for quantifying basophil activation in whole blood (40).

## Basophil Responsiveness and Clinical Phenotype

Two studies have analyzed basophil function among milk allergic subjects exhibiting different clinical phenotypes: allergic, heated milk tolerant, and outgrown. Basophil reactivity [quantified as %CD63^hi^ cells (41), or expressed as %CD63^+^ cells (42)], tested over a range of crude milk protein concentrations, was significantly lower among subjects tolerant to heated forms of milk than in those reacting to it (40). However, basophil reactivity was significantly higher in the heated milk-tolerant patients than in subjects who had outgrown milk allergy or were non-allergic. Notably, among heated milk-tolerant subjects, those with regular ingestion of heated milk exhibited less basophil reactivity, especially at lower milk protein concentrations (41). While basophil reactivity to anti-IgE stimulation was also lower in this heated milk-tolerant group, fMLP stimulation showed no differences.

Notably, Rubio et al. (43) also analyzed the value of BATs (assessed by upregulation of CD63) in distinguishing between children exhibiting persistent allergies to cow’s milk and those who had developed tolerance naturally. They developed a decisional algorithm incorporating a combination of BAT results, together with specific IgE levels and SPTs, which successfully distinguished (at the 94% level) between children who had developed tolerance naturally (these had low BAT results, as well as low specific IgE and SPT results) vs those who exhibited persistent allergies to cow’s milk. However, the most important of the three measurements used in their algorithm was the BAT result.

Taken together, these studies indicate that assessment of allergen-specific basophil responses might be a useful tool for monitoring acquisition of allergen unresponsiveness during food allergy immunotherapies.

## Measurements of Basophil Responsiveness During Oral Immunotherapy

Results of BAT assays are commonly presented as mean fluorescence intensity (MFI) of activation markers or percentage of cells that are CD63^+^ or CD63^hi^. Basophil reactivity (also known as maximal response or CDmax) and sensitivity (i.e., effective dose at 50% of the maximal activation, ED_50_ or CD_sens_) differ from patient to patient, and some studies recommend testing basophil responses over a range of allergen concentrations and expressing the results as area under the curve (AUC) of the dose response curve (44). When interpreting BAT data obtained from OIT studies it is important to keep in mind the particular representation used, as that might affect the interpretation of the data. The experimental settings that have been used to perform a BAT assay are listed in **Table 1**. The food OIT studies shown in **Table 2** list the BAT data obtained at various times during OIT treatment, and subsequent sections will discuss the clinical outcomes of some of these studies (45, 49, 51–53, 55) or the immune parameters (41, 46, 49, 51) measured. Although this review focuses on food OIT, we should note that a decrease in basophil responsiveness for food allergens has also been documented during studies of sublingual immunotherapy (SLIT) (49, 50, 57–59) and epicutaneous immunotherapy (EPIT) (60).

**Table 1:** Experimental settings that have been used to perform BAT assays.

Starting material	Whole blood (45–48) or basophil-enriched mononuclear cells (BECs) obtained after double Percoll density centrifugation (49, 50)
Anticoagulant used to collect blood	Heparin (33, 47, 51) or EDTA (49, 50, 52, 53)
Storage time and temperature	Up to 24 h at 4°C (33, 51) or within 4 h at room temperature (21, 32)
Experimental conditions	Negative Control - media with (45–47, 54) or without (51) IL-3 Single (45, 50, 52) or multiple (46–49, 51, 54, 55) concentrations of the allergen Positive Control - anti-IgE and/or fMLP
Activation conditions	30 min (45–48, 51, 52, 54) or 15 min (53) at 37°C
Frequently used activation markers	CD63 (45–48, 50, 54), CD203c (45, 46, 50, 52)

**Table 2:** BAT data obtained at various time points before, during, and after OIT in food allergy subjects.

Study size	Food extract concentration	Time points for BAT analysis	Basophil activation reported as	Major findings with respect to time into the treatment	Reference
29	Peanut (10, 1, 0.1 µg/ml)	Baseline, <4 months of OIT, 4–6 months, >6 months	%CD63hi	At a peanut concentration of 10 µg/ml, basophil activation was significantly reduced within first 4 months, and continued to decline beyond 6 months.	Jones et al. (47)
10	Egg (egg white, ovalbumin, ovomucoid)(500, 50, 5 ng/ml)	Baseline, 1 month post build-up	%CD63+, CD63 MFI	Significant decrease in CD63 expression in all patients.	Vila et al. (56)
28	Peanut (10- 10^-5 µg/ml). Egg white (1-10^-3 µg/ml).	Baseline, day 21–156, day 157–423	%CD63high, CD203c (MFI)	Significant suppression of peanut induced CD63 upregulation over time only in the peanut OIT group, no change in the placebo group. Significant reduction of CD63 upregulation at the highest egg concentration tested, the trend was evident at lower egg concentrations only in peanut OIT group. $	Thyagarajan et al. (46)
49	Peanut (1–1,000 ng/ml)	Baseline, at the time of desensitization OFC, and at the time of sustained unresponsive-ness OFC	%CD63+	Basophil responsiveness did not increase during the 4-week avoidance period between desensitization and sustained unresponsiveness.	Kulis et al. (54)
99	Peanut (0.001–100 µg/ml)	Baseline, post desensitization	AUC for %CD63+, CD63 (MFI)	No significant within-patient differences identified after treatment.	Anagnostou et al. (48)
21	Peanut (0.1, 1, 10 ng/ml)	Baseline, end of blinded escalation phase, 6 months into the maintenance phase, 12 months into maintenance phase, 6 months into continued/add on therapy, 4–6 weeks off treatment	Histamine release (% of total), CD63 (% of total basophils), CD203 (MFI), Intracellular IL-4	For subjects receiving OIT, peanut-induced histamine release and CD63 significantly suppressed at the end of dose escalation and at 6 months into maintenance, but did reverse towards the end of maintenance phase. Peanut-induced IL-4 expression significantly reduced from the end of dose escalation through maintenance compared to baseline, but increased 4–6 weeks after the subjects were taken off the therapy. #	Gorelik et al. (49)
55	Egg (0.1, 0.01 ug/ml)	Baseline, 10 months into the trial, 22 months (end of desensitization), after avoidance (24 months)	%CD63+	Basophil reactivity showed significant reduction post baseline in children receiving egg OIT compared to those receiving placebo. #	Burks et al. (55)
15	Unheated milk (100 ng/ml), heated milk (100 ng/ml)	Baseline, 12 months into the OIT	Percentage of CD63 or CD203c expression above baseline levels	#	Goldberg et al. (45)
23	Peanut (1 µg/ml)	At baseline, 3, 6, 9, 12, 18, 24, 27, and 30 months	CD203c (MFI)	Peanut induced CD203c expression in participants undergoing OIT decreased significantly at 3 months and kept reducing until 9 months compared to that in control subjects.	Syed et al. (52)
30	Peanut, Ara h 1, 2, and 6	Baseline, 3 months into active OIT, post maintenance phase (12 months), post avoidance (13 months)	AUC (%CD63hi), sensitivity (measured by using the dose that induced 50% of the maximum response)	#	Patil et al. (53)
30	Milk (10 µg/ml)	Baseline, end of build-up phase, end of maintenance, post avoidance	CD63 MFI, CD203c MFI	Spontaneous histamine release significantly reduced by week 6 in SLIT/OIT arm and remained reduced throughout (86 weeks) the study. No change in milk-induced histamine release. No change in constitutive CD63, Syk expression.	Keet et al. (50)
120	Peanut (0.1, 1, 10, 100, and 1,000 ng/ml)	Baseline, week 12, 52,104, and 117 of OIT	AUC (%CD63high), CD203c (MFI)	Peanut and anti-IgE induced AUC (%CD63hi) significantly reduced in OIT arm as early as 12 weeks and remained suppressed throughout the maintenance phase (week 104). Basophil responses were significantly lower in both arms of OIT (avoidance and low maintenance dose) at the primary endpoint (week 117), but were not significantly different vs each other. $ #	Tsai and Mukai et al. (51)

$Results further discussed in Basophil Responses and Serum Immunoglobulins.

#Results further discussed in Basophil Responses and Clinical Outcomes.

## Basophil Responses and Clinical Outcomes

In a SLIT/OIT study of 30 milk allergic patients showing favorable clinical outcomes, no reduction was observed during OIT in allergen or anti-IgE stimulated basophil histamine release, or in constitutive basophil Syk expression (50). However, spontaneous basophil histamine release was decreased in the SLIT/OIT group, beginning 20 weeks from initiating treatment. When subjects were divided based on whether or not they developed sustained unresponsiveness (passing a food challenge 6 weeks post avoidance), an increase in constitutive expression of CD63 and CD203c during the build-up phase was observed among those not developing sustained unresponsiveness. For none of the parameters, including spontaneous histamine release, did baseline values predict development of sustained unresponsiveness (50). However, this study used basophil-enriched mononuclear cells (BECs) obtained after double Percoll density centrifugation, not whole blood preparations, raising the possibility that the enrichment process might have affected the results by disrupting binding of IgG or other serum inhibitory factors (36, 37). The few studies using whole blood basophil preparations suggest that basophil responsiveness can help to predict the threshold and severity of allergic reactions during oral food challenge (43, 61, 62). In contrast, in a study of enriched basophil suspensions, Gorelik et al. (49) found no significant correlation between basophil activation (CD63 expression) and specific number or severity of allergic reactions to oral food challenges during a peanut OIT/SLIT crossover trial. They also found a negative correlation between achievement of sustained unresponsiveness (passing a food challenge at 4 to 6 weeks post avoidance) and peanut-induced histamine release, CD63 induction and IL-4 production analyzed at baseline. Histamine release and CD63 were measured with BECs after double Percoll density centrifugation and basophil intracellular IL-4 was measured in whole blood preparations. These studies suggest that processing of basophils before *ex vivo* activation can significantly affect BAT results.

Another important finding by Gorelik et al. (49) is that the significant negative correlation between basophil activation markers and development of sustained unresponsiveness was evident only at the lowest concentration of allergen extract used for *in vitro* basophil activation. This information may help in interpreting OIT results that found no significant correlations between basophil activation markers and development of sustained unresponsiveness, despite favorable clinical outcomes (50, 52). In Syed at al. (52), a single dose of peanut was used to measure activation-induced upregulation of CD203c MFI in whole blood basophils of 23 OIT subjects. Although CD203c MFI was significantly decreased in participants undergoing OIT compared to controls, there were no significant differences among those who developed unresponsiveness (no detectable clinical reaction 3 months post withdrawal of therapy) versus those who did not. Therefore, it is important to test a wide range of allergen concentrations in BATs.

The form of allergen used for *in vitro* basophil activation can also influence the results. In a peanut OIT study (53), SU (sustained unresponsiveness) and TD (transient desensitization) were evaluated in 22 participants 4 weeks post avoidance, and *in vitro* basophil responses induced by whole peanut extract or by various peanut allergen proteins were analyzed in basophils in whole blood. After desensitization by 12 months of OIT, followed by 4 weeks of peanut avoidance, basophil sensitivity (i.e., ED50, defined as the dose inducing 50% of the maximum response) to Ara h 2 significantly decreased in the SU group but not in the TD group. However, basophil sensitivity to whole peanut showed no significant difference between the groups. Moreover, when basophil reactivity was quantified as AUC, reactivity to Ara h 2 or to whole peanut was suppressed equally by the end of desensitization in both the SU and TD groups, but only the TD group rebounded post avoidance. Trends for activation with Ara h 6 were similar to those for Ara h 2, in terms of both sensitivity and reactivity (AUC).

A study of egg OIT involving 55 subjects (15 placebo, 40 OIT) tested basophil responses in whole blood and clinical desensitization at 10 and 22 months (55). Basophil responses (expressed as %CD63^+^ cells) at 10 months of OIT were significantly lower among desensitized vs non-desensitized subjects. However, no significant differences in basophil responses were observed between subjects who did or did not develop sustained unresponsiveness at 24 months (2 months after withdrawal of OIT). Notably, this study did not comment on basophil reactivity at baseline.

Different results were reported in a study of baked milk OIT in 15 milk-allergic subjects (49). Successful completion of the trial was defined as reaching the primary outcome dose of baked milk without adverse reactions at 1 year of treatment. Those succeeding in this trial exhibited a significantly lower mean difference between heated (180°C for 30 min) milk-induced and unheated milk-induced basophil CD203c expression (tested in whole blood at the beginning of the trial) than those who could not complete the trial (45). The group that successfully completed the trial also exhibited a trend toward lower values of heated (hypoallergenic form) milk-driven minus unheated (hyper-allergenic form) milk-driven basophil CD63 expression. This study suggested that patients whose basophils reacted less to the hypoallergenic vs more allergenic forms of this antigen at baseline became desensitized to the hypoallergenic form by the end of the trial.

**Figure 1:**
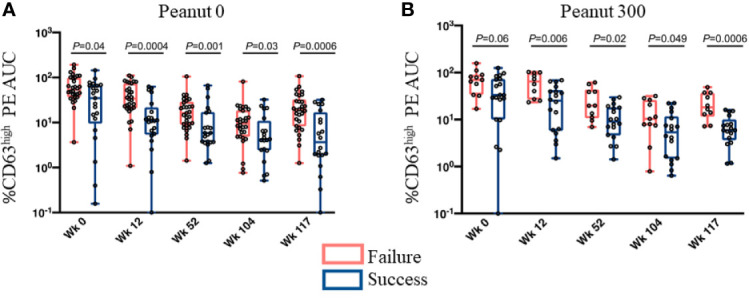
Basophil responsiveness in OIT treatment outcome groups (i.e., Success vs Failure) is significantly different at all times tested. Basophil responsiveness to peanut extract (%CD63^high^ PE AUC) evaluated at multiple time points during OIT. Subjects were divided into two groups based on whether they did (Success) or did not (Failure) develop sustained unresponsiveness assessed by an oral food challenge at week 117. **(A)** Peanut 0 – the treatment arm in which subjects completely avoided peanut consumption after the end of desensitization phase (Week 104). **(B)** Peanut 300 – the treatment arm that maintained subjects at a low dose (i.e., 300 mg/day) of peanut consumption, from week 104 onwards. Whiskers represent the range (minimum to maximum values of AUC), boxes extend from 25^th^ to 75^th^ percentiles. The lines in the middle of the boxes are medians. Individual values are shown as circles. P values were determined by Mann-Whitney test. These are from Figures 4A, B of Tsai and Mukai et al. (51).

Finally, our study of peanut OIT (51) divided 120 participants (25 placebo, 95 OIT) into groups depending on whether or not they developed sustained unresponsiveness (passing the oral food challenge either 13 weeks post avoidance or after 13 weeks of a low maintenance dose consumption, i.e. week 117 of OIT). A retrospective analysis of the groups with or without sustained unresponsiveness revealed that peanut-specific basophil responses were significantly lower among the group that developed sustained unresponsiveness at all time-points (weeks 12, 52, 104, 117) tested during OIT, and also at baseline (**Figure 1**).

**Figure 2:**
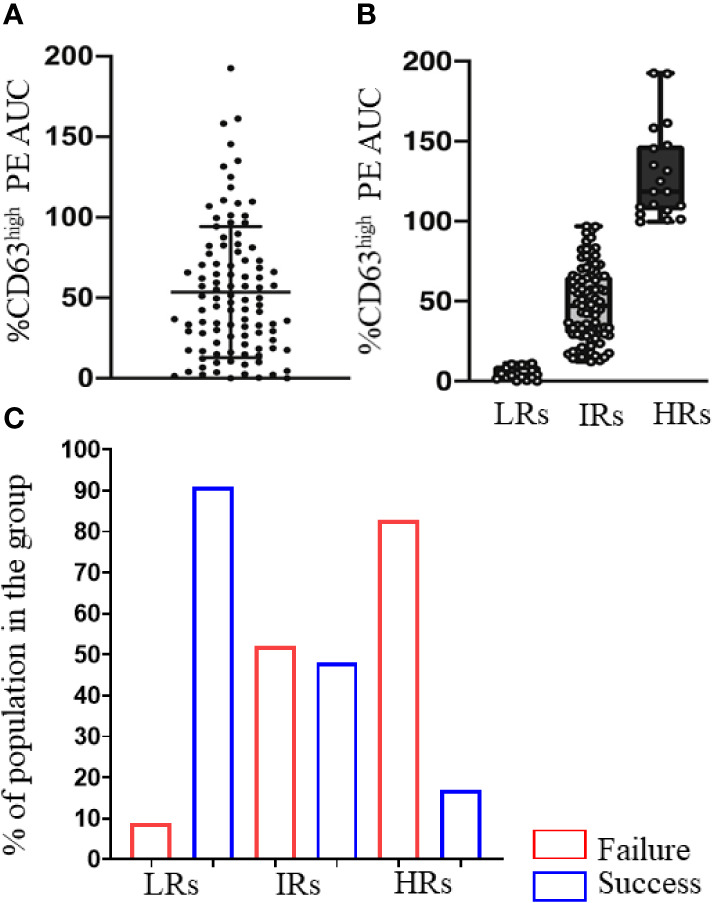
Low basophil activation at week 0 is associated with sustained unresponsiveness after OIT. **(A)** Peanut-induced %CD63^high^ basophils (%CD63^high^ PE AUC) in the 120 participants at baseline. Scatter dot plot with ± SD. **(B)** Peanut-induced %CD63^high^ basophils of LRs (PE AUC < 12.09), IRs (PE AUC >12.09 and <97.37) and HRs (PE AUC >97.37). Individual values are shown as circles. **(C)** Percentage of the subjects in LR, IR, HR groups that showed sustained unresponsiveness (Success) or not (Failure) at week 117, assessed by an oral food challenge. These are from Figures 6A, B and Table 1 of Tsai and Mukai et al. (51).

Notably, we (51) also grouped peanut OIT participants according to baseline basophil responsiveness to peanut (calculated as AUC for %CD63^high^ cells) into LR (low responders), IR (intermediate responders) and HR (high responders) (**Figures 2A, B**). We found that LRs tolerated 2–3 times more peanut protein at the time of enrollment, pointing towards a relationship between basophil responsiveness and severity of allergic reactions during food challenges. Furthermore, while a larger fraction of the LR group (91%) developed sustained unresponsiveness at the primary endpoint (**Figure 2C**), those subjects from the IR and HR groups who showed substantial reduction (80–90%) of their peanut-induced basophil responses during the trial also achieved sustained unresponsiveness. This analysis thus revealed two groups of subjects that achieved sustained unresponsiveness post OIT - allergic patients with mild antigen-specific basophil responsiveness at the beginning of the trial and patients who undergo significant reduction of allergen specific basophil responses due to OIT.

## Basophil Responses Among “Releaser” and “Non-Releaser” Basophils

Several studies have shown that blood basophils from some individuals fail to degranulate upon stimulation through the FcϵRI (63–72). These basophils have been widely called “non-releasers” (64–71) or sometimes “anergic” (72), depending on the study. During a year-long study by Kepley et al. (68), there were three “non-atopic, non-releasers”, defined as patients whose basophils failed to release histamine in response to anti-IgE antibody (calculated using basophil-enriched cell populations isolated by Percoll gradient centrifugation yielding 25–60% basophil purity). However, the basophils from one of these subjects converted into “releasers”. As “releasers”, these basophils had detectable Syk protein expression (analyzed in western blots performed with >99% pure basophils obtained by sequential positive and negative selection and flow sorting) that was undetectable in basophils obtained during the “non-releaser” phase in the same patient.

A study by Puan et al. (72) divided individuals according to the functional state of their whole blood basophils. HDM^R^ (house dust mite responders) had CD63^+^ basophils after HDM stimulation (using an empirically determined threshold of at least 38% CD63^+^ cells). HDM^NR^ (HDM non-responders) were defined as giving no response to HDM allergens but at least 38% CD63^+^ basophils after anti-FcϵRI stimulation. They defined “anergic” basophils as those that responded neither to HDM allergens nor to anti-FcϵRI stimulation. For 38 individuals, the functional state of their basophils was defined at two time points separated by a period of approximately 2 years. While 26 of these 38 individuals remained in the same functional state, 13 underwent transitions between one of the reactive states (HDM^R^ or HDM^NR^) and the anergic state. Moreover, such transitions happened in both directions. Conversion between releaser and non-releaser phenotype was also noted by Youseff et al. (71), in a four year study. This study categorized individuals as non-releasers if their basophils, obtained as basophil-enriched cell populations (1–55% basophil purity) by Percoll gradient centrifugation of anti-coagulated blood, released less than 12.7% histamine, a cut-off decided by applying statistical methods to the entire data set. Youseff et al. (71) found that 8 of 8 asthmatic non-releasers (13% of the asthmatic subjects enrolled in the study) and 16 of 23 control non-releasers (28% of control subjects enrolled), converted to releaser status at least once over the course of the study.

Overall, each of these studies suggests that basophils from individual donors may be able to cycle in and out of responsiveness over time. Interestingly, Yuoseff et al. (71) observed that the presence of non-releaser basophils does not rule out the diagnosis of asthma. By contrast, Puan et al. (72) suggested that anergic individuals are less likely to develop atopy (assessed by HDM SPTs) and symptoms of allergic rhinitis than those who responded to HDM (i.e., HDM^R^). It therefore will be interesting to investigate further, in different diseases, the potential relationships between fluctuations in the functional state of basophils during OIT, or other clinical interventions, and overall clinical outcomes.

## Basophil Responses and Serum Immunoglobulins

A study comparing basophil responses among clinical phenotypes characterized as allergic, heat-denatured milk tolerant and outgrown (41), noted a strong correlation between basophil responses (quantified as %CD63^hi^ cells) and specific IgE levels among all groups. Since then, many OIT studies have recorded longitudinal changes in the levels of serum antibodies during OIT, but very few comment on correlations between such changes and basophil responses.

A peanut OIT study involving 28 subjects recorded basophil responses in whole blood at various times (day 0, days 21–156 and day 157–423) (46). Compared to baseline, peanut OIT resulted in significantly lower peanut-induced basophil responses (% CD63^high^) for all 6 concentrations of peanut used for *in vitro* activation. This coincided with significantly increased peanut-specific IgG4 levels. Interestingly, in peanut-egg dual allergic subjects (9 among the 28 enrolled), basophil responses to egg were also decreased. However, a significant reduction in basophil responses was only detected with the highest of four tested concentrations of egg extract. There were no changes in egg-specific IgG4, which might be the reason for this mild effect.

In another peanut OIT study involving 21 subjects (49), baseline levels of basophil IL-4 expression (quantified using basophils in whole blood) in response to all three doses of peanut used for *in vitro* activation was positively correlated with peanut-specific serum IgE levels. However, when peanut-induced basophil CD63 and histamine release were evaluated using BECs obtained after double Percoll density centrifugation, no correlation with any serum antibody levels was detected. These results thus may have been influenced by studying basophils in whole blood vs post enrichment.

We studied peanut OIT in 120 participants, testing basophils in whole blood (51). We analyzed the relationship between basophil responses and serum antibody levels at baseline (week 0) and at the primary endpoint (week 117). At both time points, peanut-induced basophil responses (calculated as AUC for %CD63^high^ cells) showed weakly significant positive correlations with levels of serum IgE against peanut and peanut-components (e.g., Ara h 1, Ara h 2 and Ara h 3) and with the specific IgE/total IgE ratio, and a negative correlation with the specific IgG4/specific IgE ratio. We noted no significant correlations of basophil activation with levels of specific IgG4. A similar conclusion was supported when we classified our participants, according to basophil responsiveness at baseline, into LR (low responders), IR (intermediate responders) and HR (high responders) (51). Thus, at the time of enrollment, the LR group differed from the IR or HR subjects not only in having lower peanut-specific and component-specific IgE levels and a smaller ratio of specific IgE/total IgE, but also in having a higher ratio of specific IgG4/specific IgE.

These observations suggest that basophil responses to allergens reflect the coordinated actions of both activating and inhibitory immunoglobulins—and that the proportion of such immunoglobulins is more critical than their absolute levels. However, the proportion of immunoglobulins does not take into account their relative avidity, their affinity, or the epitopes they recognize. These also are important factors that must be considered, in addition to ratios of activating/inhibitory immunoglobulins, when interpreting basophil responses.

## Tissue Mast Cells and Oral Imunotherapy

Compared to blood basophils, it has been difficult to evaluate mast cell populations that may participate in food allergy. However, food allergy studies in mice have described the critical role played by mucosal mast cells, both in acquisition of susceptibility towards food allergens and in their contribution to the severity of the allergic reactions (3, 4). Duodenal biopsies from food allergic patients have also showed enhanced expression of mast cell-associated transcripts compared to control subjects (3).

Nevertheless, many challenges have hindered the detailed study of human mucosal mast cells at the site of the disease (i.e., GI tissues). Examples of such problems include the sparse distribution of gut mast cells, making it difficult to obtain sufficient numbers for many studies. Indeed, isolation of these cells for *in vitro* analysis requires enzymatic and mechanical tissue dispersion, processes that likely change the intrinsic activities of the cells. Moreover, *in vitro* studies may not recapitulate mast cell actions *in situ*, as these cells’ responses to microenvironmental cues can change their phenotype and function (73, 74). Due to these limitations, it has become common to evaluate instead the responses of skin mast cells, which can be conveniently studied *in situ* (5).

Indeed, whenever IgE-mediated food allergy is suspected, SPTs are commonly recommended to identify the causative allergen, along with measurements of serum levels of allergen-specific IgE. SPTs provoke allergen-mediated mast cell degranulation in the skin, leading to measurable wheal-and-flare reactions. For some common food allergens, wheal size thresholds have helped to confirm sensitization and to indicate a high probability of food allergy (75, 76). However, although SPT diameters differ between allergic and non-allergic subjects, they might not distinguish subjects that have naturally outgrown allergy from those who are still allergic (41, 42). It also should be remembered that skin mast cells live for months or longer and take weeks to change responsiveness to antigens recognized by cell-bound IgE, while mature blood basophils live only for days (7, 10). Such considerations suggest that losses in skin responses to allergens might occur substantially slower than the development of clinical unresponsiveness, which may be more reflective of changes in basophils or, perhaps, gastrointestinal mast cells.

The few studies of food OIT (47, 50, 52, 55, 77) and SLIT (50, 57–59, 78) containing longitudinal analyses of SPT diameters note their reduction, either during or toward the end of the study. In analyzing peanut OIT in 29 participants, Jones et al. found SPT diameters began to significantly decrease beginning at 6 months into the trial (47). This study did not discuss the correlations between the SPT results and the clinical outcomes. However, just 3 months into the OIT, peanut-specific IgG and IgG4 increased significantly, followed by significant suppression of basophil responses (measured as %CD63^bright^ for individual peanut concentrations) 4 months into the trial (47). In another study of peanut OIT involving 23 participants (52), basophil responses and SPT results followed a similar trend, but with different kinetics of reduction: peanut-induced CD203c MFI was significantly reduced in OIT subjects vs controls at 3 months into therapy, whereas SPT diameters significantly diminished at 12 months. Nevertheless, both basophil responses (assessed with a single dose of food allergen) and SPT results did not significantly differ among subjects developing sustained unresponsiveness (at 3 months post therapy withdrawal) vs those who did not.

Another study monitoring the response to Omalizumab in 14 peanut-allergic subjects noticed a significant reduction (more than 80% from baseline) in peanut-induced histamine release AUC in 5 subjects within 8 weeks of initiating therapy (16). These five did not have reduced SPT responses this early in the treatment. In the other nine subjects, peanut-induced basophil histamine release AUC was unchanged by week 8 but 10-fold more peanut allergen was required to induce the maximal histamine release. Although this shift in basophil sensitivity was smaller than the treatment-induced improvement (approximately 50 fold) in the dose of allergen needed to induce a clinical response, there was a temporal association between basophil dose response and clinical response as early as week 8 of treatment. However, SPT responses did diminish by the end of treatment (week 24).

**Figure 3:**
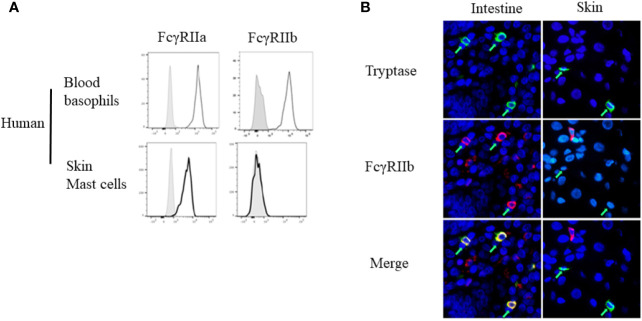
Differential expression of Fcγ receptor (FcγR)IIb by basophils and different mast cell populations. **(A)** FcγRIIa and FcγRIIb expression by human peripheral blood basophils and human Skin mast cells, assessed by flow cytometry. **(B)** FCγRIIb expression by human intestinal or skin mast cells, assessed by immunofluorescence staining for mast cell tryptase (green) and FcγRIIb (red) of human tissue sections. Mast cells are indicated by green arrows. These are from Figure 1A, and Figure 3 of Burton et al. (90) with permission.

Overall, studies of OIT indicate that progressive desensitization evolves much faster than the changes in skin mast cell reactivity. Another probable explanation reflects the mechanism of OIT-induced desensitization. Data from milk- or egg-allergic subjects showed that food-specific IgG levels not only exhibit an inverse correlation with the reaction severity but also increase in parallel with natural resolution of the allergy (79, 80). Both OIT and early food introduction strategies elicit food-specific IgG antibodies (47, 57, 81–83). These then can act through inhibitory Fcγ receptors (i.e., FcγRIIb) to inhibit IgE-FcϵRI mediated hypersensitivity (4). Studies of both mouse (84–87) and human (88) mast cells and basophils (36, 37) have provided evidence for this counter-regulatory mechanism. This also may explain why some patients with food-specific IgE can safely ingest food with no reaction and why the presence of allergen-specific IgE is needed but not sufficient to induce a clinical reaction (89). Notably, human skin mast cells do not ordinarily express the inhibitory receptor FcγRIIb (**Figure 3A**) (90, 91). It therefore seems very likely that this counter-regulatory mechanism is ineffective in skin mast cells.

Burton et al. have demonstrated the expression of FcγRIIb on human mast cells throughout the gastrointestinal tract, including the tongue, esophagus, small and large intestine, and (weakly) in the stomach (90). Using a humanized mouse model, they also tested the physiologic role of the IgG-FcγRIIb pathway in suppression of IgE-triggered systemic anaphylaxis, evidence that the receptor was functional (90). These observations highlight the heterogeneity among tissue mast cells (**Figure 3B**). They also provide an explanation for the relative contribution of FcγRIIb in suppressing hypersensitivity in mice vs humans.

**Figure 4:**
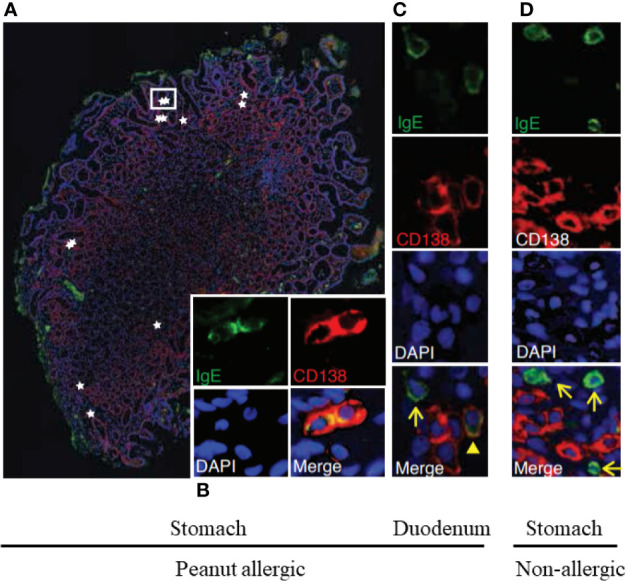
Stomach and duodenum are reservoirs of IgE^+^ B lineage cells in peanut allergic people. Immunofluorescence of stomach **(A, B)** and duodenal **(C)** biopsies from peanut allergic donors or from non-allergic stomach **(D)**. IgE (green), plasma cell marker CD138 (red), and nuclei (DAPI; blue) **(A)**. IgE^+^CD138^+^plasma cells (stars) localized singly and in clusters between gastric glands; a white rectangle outlines two IgE^+^CD138^+^plasma cells, for which single-channel staining is shown in **(B)**. **(C)** IgE^+^CD138^+^ plasma cell (arrowhead) and IgE^+^CD138^−^ putative mast cell (arrow). **(D)** Representative image from a non-allergic donor. IgE^+^CD138^−^ putative mast cells were observed (arrows), but IgE^+^CD138^+^ plasma cells were absent. These are from Figures 1E–H of Hoh and Joshi et al. (92).

A study by Hoh and Joshi, et al. provides yet another perspective into the events taking place in the GI tract of allergic subjects (92). By performing high throughput DNA sequencing on biopsies of esophagus, stomach, and duodenum from peanut-allergic patients and controls, they found that peanut-allergic patients harbored large numbers of somatically-mutated, clonally-expanded, allergen-specific IgE^+^ B lineage cells, including cells with a plasma cell phenotype, in their GI tissues (**Figure 4**). Furthermore, the co-occurrence of IgE-expressing and non-IgE-expressing clonally-related B lineage cells in the same biopsy samples indicated local isotype switching. Common convergent heavy chain sequences shared between allergic donors suggested that common immunoglobulin genetic rearrangements contributed to the pathogenesis of the disease.

Such IgE^+^ plasma cells present throughout the GI tract of allergic subjects could disproportionately contribute to the FcϵRI bound IgE on mast cells present at the same locations (**Figure 4**). Regional differences in local IgE^+^ plasma cell clones might lead to differences in local mast cell IgE loading, contributing to the clinical heterogeneity of patient symptoms and thresholds post allergen exposure. Stomach and duodenal IgE^+^ clone counts in allergic patients correlated with peanut allergen-specific IgE levels in serum, where they will affect responses of circulating blood basophils. Indeed, basophils may be the first responders to food antigens that gain access to the blood. However, it will be interesting to determine whether the effects of OIT on gut mucosal mast cells may actually precede those influencing mast cells present in the skin. Specifically, it is possible that reductions in the reactivity of basophils and gut mast cells during OIT will exhibit more similar kinetics than the later reduction observed in the responses of skin mast cells.

## Conclusions

Basophil responses measured *ex vivo* before, during and after immunotherapy can help to differentiate between transient desensitization and sustained unresponsiveness. Moreover, considerations such as study size, evaluation of basophil activation in whole blood vs in enriched preparations, the range of antigen dosages used, and which basophil responses are assessed (e.g., %CD63^+^ vs AUC of %CD63^+^ vs CD203c MFI, etc.) may critically influence the information gained from a study. For testing relatively large numbers of specimens that may originate at significant distances from the test site, we currently favor the approach described in **Table 3**. However, clearly, more investigations are required to establish which type of BAT measurements, and which BAT thresholds, can identify those allergic subjects who can benefit most from OIT. Finally, an important role for mucosal mast cells in food allergy has been suggested by work in both humans and mice. For example, our high throughput DNA sequencing study revealed that the gastrointestinal tract of food allergic patients is a reservoir of IgE^+^ B lineage cells. This finding emphasizes the need to study, in particular, those gastrointestinal mast cells that are near such IgE-producing plasma cells.

**Table 3:** Approach for testing relatively large number of BAT specimens that originate at significant distances from the testing site.

Starting material	Whole blood
Anticoagulant used	Heparin
Storage time and temperature	Up to 4 h at room temperature or up to 24 h at 4°C
Experimental conditions	Negative control (RPMI) 5 Concentrations of allergen - e.g., 0.1, 1, 10, 100, 1,000 ng/ml of Peanut extract Positive controls - anti-IgE (1 µg/ml) and IL-3 (2 ng/ml) (tested separately)
Activation conditions	30 min at 37°C
Gating strategy	CD123+HLA-DR-
Activation markers evaluated	CD63 (%CD63high), CD203c (ΔCD203c MFI)

If BATs are to be performed on a large number of samples obtained at a significant distance from the testing site, we favor immediately bringing the blood to 4°C and then keeping the blood at that temperature for ~24 h before testing (33,51). This keeps the storage conditions uniform for all of the samples being evaluated.

## Author Contributions

AP wrote most of the text, with help from SG. All authors participated in the editing, and oversight of the content, of the text. All authors contributed to the article and approved the submitted version.

## Conflict of Interest

SG reports grants from NIH. RC reports grants from NIAID, CoFAR, Aimmune, DBV Technologies, Astellas, Regeneron, is an Advisory member for Alladapt, Genentech, Novartis, and receives personal fees from Before Brands outside the submitted work. KN reports grants from NIAID, NHLBI, NIEHS, Food Allergy Research & Education (FARE); other funding from World Allergy Organization (WAO), Cour Pharma, Before Brands, Alladapt, Latitude, IgGenix, Immune Tolerance Network (ITN), and NIH clinical research centers outside the submitted work. In addition, KN has the following patents pending: Inhibition of Allergic Reaction to Peanut Allergen using an IL-33 Inhibitor; Special Oral Formula for Decreasing Food Allergy Risk and Treatment for Food Allergy; Basophil Activation Based Diagnostic Allergy Test; Granulocyte-based methods for detecting and monitoring immune system disorders; Methods and Assays for Detecting and Quantifying Pure Subpopulations of White Blood Cells in Immune System Disorders; Mixed Allergen Compositions and Methods for Using the Same; Microfluidic Device and Diagnostic Methods for Allergy Testing Based on Detection of Basophil Activation. SB has consulted for Regeneron, Sanofi and Novartis on topics unrelated to this study, owns shares in AbCellera and CareDx, and has patents awarded or submitted related to immunoglobulin gene and protein analysis.

The remaining authors declare that the research was conducted in the absence of any commercial or financial relationships that could be construed as a potential conflict of interest.

The reviewer EK declared a past co-authorship with several of the authors RSC and KN to the handling editor.
